# Pro-inflammatory diet association with sudden sensorineural hearing loss: a case-control study

**DOI:** 10.1017/S0022215126105416

**Published:** 2026-09

**Authors:** Kadir Sinasi Bulut, Basak Yalciner, Burak Celik, Esra Zeynep Karakus, Erva Nur Topaloglu

**Affiliations:** 1 Otolaryngology, Head and Neck Surgery, Ankara Yildirim Beyazit University, Türkiye; 2 Otolaryngology, Head and Neck Surgery, Medical Park Hospitals Group, Türkiye; 3 Otolaryngology, Head and Neck Surgery, Ministry of Health Ankara City Hospital, Türkiye

**Keywords:** diet, hearing loss, sudden, inflammation, nutrition assessment

## Abstract

**Objectives:**

This study investigated whether the inflammatory potential of diet, assessed by the Dietary Inflammatory Index, is associated with sudden sensorineural hearing loss by comparing patients with sudden sensorineural hearing loss and healthy controls.

**Methods:**

This retrospective case–control study included 30 patients with sudden sensorineural hearing loss and 32 matched controls. Dietary intake in the preceding 12 months was assessed by food frequency questionnaire, and Dietary Inflammatory Index scores were calculated. Between-group comparisons and receiver operating characteristics analysis were performed.

**Results:**

Mean Dietary Inflammatory Index score was significantly higher in the sudden sensorineural hearing loss group than in controls (2.27 ± 1.21 vs. 1.46 ± 1.14; *p* = 0.01). Patients with sudden sensorineural hearing loss had higher energy, saturated fat and cholesterol intake, while omega-3 fatty acid and fibre intake were lower (*p* < 0.05). Dietary Inflammatory Index scores demonstrated moderate discriminative ability for sudden sensorineural hearing loss (area under the curve = 0.690; *p* = 0.005).

**Conclusion:**

A pro-inflammatory dietary profile may be associated with increased sudden sensorineural hearing loss risk and may contribute to understanding modifiable risk factors.

## Introduction

Sudden sensorineural hearing loss (SSNHL) is characterised by a rapid decrease in hearing of at least 30 dB affecting three consecutive frequencies within a 72-hour period and is considered an otologic emergency.^1^ The annual incidence is estimated at 5–27 per 100,000 individuals, and patients may experience tinnitus, vertigo and anxiety related to hearing impairment.^2^ In nearly 90 per cent of cases, the aetiology cannot be determined. Suggested mechanisms include viral, vascular and autoimmune processes as well as membrane-related cochlear injury.^3^

Dietary patterns dominated by processed foods and saturated fats tend to promote inflammation, whereas diets rich in plant-based foods and omega-3 fatty acids have anti-inflammatory effects.^4^ The Dietary Inflammatory Index (DII) was introduced by Shivappa *et al.* to quantify the inflammatory potential of overall dietary intake.^5^ This index integrates multiple nutrients and food components to classify diets along a spectrum from anti-inflammatory to pro-inflammatory. Lower scores reflect anti-inflammatory dietary patterns, whereas higher scores indicate pro-inflammatory patterns.^5^ Higher DII values have been associated with several chronic conditions, including metabolic and cardiovascular disorders.^6^^,^^7^

Increasing evidence suggests that nutritional factors influence auditory function, and antioxidant vitamins together with omega-3 fatty acids may protect against hearing loss.^8^^,^^9^ However, the overall dietary inflammatory profile has not yet been specifically investigated in sudden hearing loss.

Therefore, this study aimed to evaluate the association between dietary inflammatory potential and SSNHL by comparing DII scores between patients and healthy controls.

## Materials and methods

### Study population

This study was designed as a retrospective case–control study. The study included 30 patients diagnosed with SSNHL at an otorhinolaryngology clinic between January 2025 and November 2025. Patients were invited to participate in the study via telephone contact or face-to-face interviews during November and December 2025, and dietary assessments were conducted. The control group consisted of 32 healthy volunteers matched to the patient group for age and sex. Control participants were recruited from individuals who presented to the same institution for routine health checkups.

Patients in the SSNHL group were adults with unilateral sudden sensorineural hearing loss (≥30 dB across three consecutive frequencies within 72 hours) confirmed by pure-tone audiometry. Control participants were age-matched adults without hearing loss or systemic disease. Exclusion criteria included bilateral hearing loss, known inner ear or chronic otologic disease, recent head trauma or ototoxic drug use, autoimmune or malignant disease, major surgery within the past six months, pregnancy or lactation, inability to complete dietary assessment or adherence to special or anti-inflammatory diets.

### Data collection

Age, gender, height, body weight and comorbid diseases were recorded for all participants. The body mass index (BMI) for each participant was calculated as their body weight in kilograms divided by their height in metres squared.

### Dietary assessment and DII calculation

Dietary intake during the 12 months preceding SSNHL diagnosis was assessed using a food frequency questionnaire administered via face-to-face or telephone interviews. The questionnaire evaluated consumption frequency and portion sizes of commonly consumed foods, categorised from “never/rarely” to “four or more times per day.” Portion sizes were estimated using standard household measures and photographic portion guides. Nutrient and energy intakes were calculated using a nationally validated food composition database, and household measures were converted into grams using standardised portion weights.

DII scores were calculated using R software (version 4.5.2) according to the methodology of Shivappa *et al*.^5^ The calculation included parameters of energy; carbohydrate; protein; total fat; saturated fat; monounsaturated fatty acids; polyunsaturated fatty acids; omega-3 and omega-6 fatty acids; cholesterol; fibre; vitamins A, B6, B12, C, D and E; iron; magnesium; and zinc. Negative DII scores indicate an anti-inflammatory diet, while positive scores represent a pro-inflammatory diet.

### Statistical analysis

IBM SPSS Statistics 25.0 (IBM Corp., Armonk, NY, USA) was used to conduct statistical analyses. R software (version 4.5.2) was used to perform DII calculations. The Shapiro–Wilk test was used to determine if continuous variables were normal. To compare the two groups, we utilised independent samples *t*-test for normally distributed data, presenting these variables as mean ± standard deviation. For data that violated normality assumptions, the Mann–Whitney *U*-test was applied, with results expressed as median (interquartile range). The chi-squared test was used to examine categorical variables that were shown as numbers and percentages. Area under the curve (AUC), sensitivity and specificity values were computed using receiver operating characteristic (ROC) curve analysis to assess the discriminative potential of the DII score in predicting SSNHL. Post-hoc power analysis was performed to determine the statistical power of the study using GPower 3.1(Heinrich Heine University, Düsseldorf, Germany) software. Power calculation was conducted using non-central t-distribution for independent samples t-test with α = 0.05 (two-tailed). A *p*-value less than 0.05 was considered statistically significant.

## Results

A total of 62 participants were included: 30 patients with SSNHL and 32 controls. The groups were comparable in terms of age, sex distribution and body mass index (Table 1).

**Table 1.** Demographic and anthropometric characteristics of the patient and control groups Table 1 long description.

Parameter		Sudden hearing loss group (*n* = 30)	Control group (*n* = 32)	*p*-Value
Age, y		42.60 ± 12.47	42.25 ± 13.33	0.915
Sex, n (%)				0.585
	Male	18 (60.0)	17 (53.1)	
	Female	12 (40.0)	15 (46.9)	
BMI (kg/m^2^)		24.31 ± 4.11	23.97 ± 3.61	0.731

BMI = body mass index.

The distribution of comorbidities in the patient and control groups was evaluated. The most frequently reported comorbidities in both groups were hypertension and diabetes mellitus. No statistically significant difference was found between groups in terms of presence and distribution of comorbidities.

The DII score was significantly higher in the SSNHL group than in controls, indicating a more pro-inflammatory dietary pattern among patients (*p* = 0.01).

Analysis of macronutrient intake demonstrated that patients with SSNHL consumed more total energy and carbohydrates than controls. In addition, saturated fat and cholesterol intake were higher in the patient group, whereas omega-3 fatty acid and dietary fibre intake were lower. No significant differences were observed for total fat, monounsaturated fatty acids, omega-6 fatty acids, protein intake or vitamin B6 and B12 levels (Table 2).

**Table 2.** Comparison of DII score, macronutrients, micronutrients and vitamins–minerals between groups Table 2 long description.

	Sudden hearing loss group	Control group	
	(*n* = 30)	(*n* = 32)	
Parameter	Mean ± SD	Mean ± SD	*p*-Value
DII score	2.27 ± 1.21	1.46 ± 1.14	**0.01**
Energy, *kcal*	2251.59 ± 294.28	2076.49 ± 316.11	**0.028**
Carbohydrate, *g*	306.33 ± 54.02	277.34 ± 43.71	**0.023**
Protein, *g*	73.08 ± 12.66	67.82 ± 12.52	0.106
Total fat, *g*	81.55 ± 17.88	77.32 ± 16.52	0.31*
Saturated fat, *g*	28.61 ± 7.83	23.03 ± 8.85	0.035*
MUFA, *g*	33.98 ± 7.32	35.40 ± 10.24	0.532
Fibre, *g*	17.37 ± 7.79	25.50 ± 8.16	**<0.001**
Cholesterol, m*g*	355.67 ± 90.27	257.66 ± 98.91	**<0.001**
Omega−3, *g*	0.65 ± 0.45	1.00 ± 0.63	0.027*
Omega−6, *g*	16.87 ± 5.42	17.38 ± 5.49	0.646*
Vitamin A, *µg*	948.33 ± 308.55	709.38 ± 195.15	0.004*
Vitamin C, *mg*	93.17 ± 17.59	80.56 ± 19.47	**0.01**
Vitamin E, *mg*	10.18 ± 2.59	7.45 ± 2.66	**<0.001**
Vitamin B6, *mg*	1.51 ± 0.27	1.49 ± 0.25	0.771
Vitamin B12, *µg*	4.81 ± 0.93	5.77 ± 2.46	0.326*
Folate, *µg*	334.33 ± 104.34	215.0 ± 100.25	**<0.001**
Iron, *mg*	15.47 ± 4.18	19.59 ± 4.56	<0.001*
Magnesium, *mg*	309.17 ± 47.92	266.88 ± 58.44	**0.03**
Zinc, *mg*	12.03 ± 3.15	13.53 ± 2.65	0.018*

DII = Dietary Inflammatory Index; MUFA, monounsaturated fatty acids.

*Note:* Mann–Whitney *U*-test

(*) was used for non-normally distributed variables.

Regarding micronutrients, the distribution of most vitamins and minerals did not differ consistently between groups, suggesting that overall dietary inflammatory profile rather than single nutrient deficiency was associated with SSNHL.

**Figure 1.**
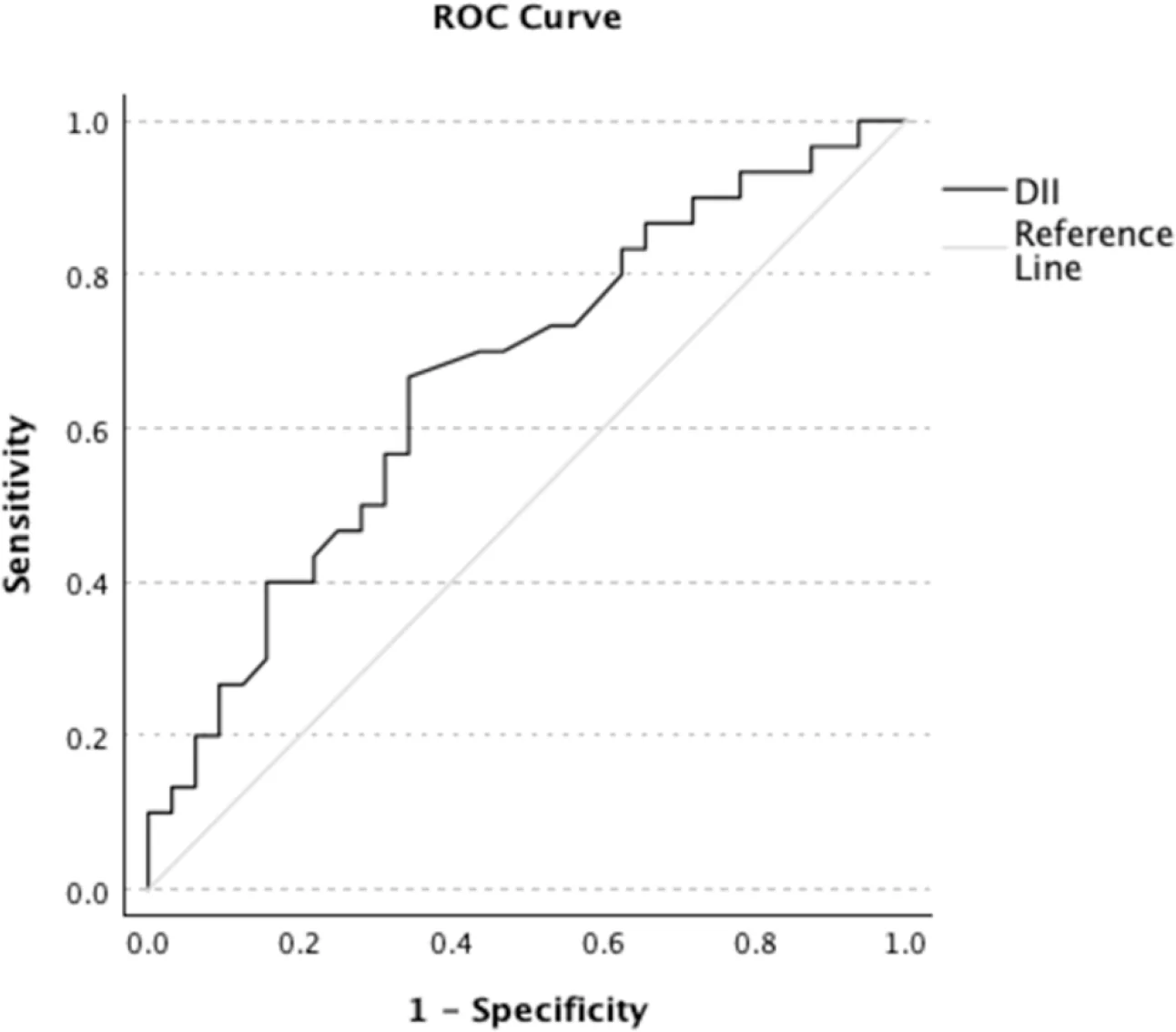
ROC curve analysis of DII and daily energy intake for predicting SSNHL. DII = Dietary Inflammatory Index; ROC = receiver operating characteristic; SSNHL = sudden sensorineural hearing loss.

ROC curve analysis demonstrated that the DII score had moderate discriminative ability for SSNHL (AUC = 0.690; 95 per cent confidence interval [CI]: 0.557-0.822; *p* = 0.005) At a cutoff value of 1.60, the DII score showed a sensitivity of 70 per cent and specificity of 66 per cent for predicting SSNHL (Fig. 1). Post-hoc power analysis revealed that the study achieved a statistical power of 76.13 per cent (1-β = 0.7613) with a medium effect size (Cohen’s d = 0.6897). This power level indicates that the study had acceptable ability to detect the observed difference in DII scores between SSNHL patients and controls.

## Discussion

This study is the first to systematically evaluate the relationship between SSNHL and dietary inflammatory potential. Previous studies have shown that higher DII scores are associated with increased adiposity and metabolic alterations in neuroinflammatory diseases such as multiple sclerosis, highlighting the role of dietary inflammatory potential in neurological conditions.^10^ The principal finding of our study is that the DII score was significantly higher in SSNHL patients compared to healthy controls (2.27 ± 1.21 vs. 1.46 ± 1.14; *p* = 0.01). This finding suggests that a pro-inflammatory diet may play a role in the development of SSNHL. Additionally, saturated fat and cholesterol intake were found to be high, while omega-3 fatty acid and dietary fibre intake were low in SSNHL patients. ROC analysis demonstrated that DII score had moderate discriminative power in predicting SSNHL (AUC: 0.690; *p* = 0.005).

The relationship between dietary inflammatory potential and hearing loss has gained increasing attention in recent years. Wang *et al*. investigated the association between DII and sensorineural hearing loss (SNHL) using National Health and Nutrition Examination Survey (NHANES) data.^11^ Smooth curve fitting analysis demonstrated that SNHL risk increased when DII score exceeded 5.15. Through Mendelian randomisation analysis, they found that fish consumption significantly reduced SNHL risk and that six specific gut microbiota species showed significant causal associations with SNHL, supporting the “gut–inner ear axis” hypothesis. Similarly, Fu *et al*. detected an inverted U-shaped relationship between Energy-adjusted DII (E-DII) score and low-frequency hearing loss.^12^ Our study proposes that this inflammatory state can be modulated through diet and is consistent with these current findings in the literature.

The mechanism by which a pro-inflammatory diet leads to SSNHL is multifactorial. Diets high in saturated fat and refined carbohydrates promote a systemic inflammatory state characterised by elevated circulating cytokines.^4^^,^^13^ This inflammatory response may impair cochlear microcirculation through endothelial dysfunction.^14^^,^^15^ Experimental models have shown that inhibition of interleukin-6 (IL-6) signalling can attenuate inflammation-related cochlear damage.^16^

In our study, the finding that omega-3 fatty acid intake was 35 per cent lower in SSNHL patients compared to the control group (0.65 g vs. 1.00 g; *p* = 0.027) is noteworthy. Omega-3 fatty acids are known to exert anti-inflammatory and neuroprotective effects.^17^ In a prospective cohort study by Curhan *et al.* involving more than 65,000 women, higher intake of fish and omega-3 fatty acids has been associated with a reduced risk of hearing loss.^8^ In another prospective cohort study by the same research group, consuming fish at least twice weekly has been linked to a lower incidence of age-related hearing loss.^18^ Experimental studies have demonstrated that omega-3 polyunsaturated fatty acids exert protective effects on cochlear function and slow the progression of hearing loss in animal models, possibly through anti-inflammatory and tissue-protective mechanisms.^19^ Our findings suggest that omega-3 deficiency may be a potential risk factor for SSNHL.

The findings that saturated fat intake was 24 per cent higher (28.61 g vs. 23.03 g; *p* = 0.035) and cholesterol intake was 38 per cent increased (355.67 mg vs. 257.66 mg; *p* < 0.001) in the SSNHL group are significant. Saturated fatty acids activate Toll-like receptor 4 signalling, leading to nuclear factor kappa-light-chain-enhancer of activated B cells (NF-κB) -mediated inflammatory cytokine release.^20^ Furthermore, excessive saturated fat intake may compromise microvascular circulation via endothelial dysfunction.^21^ Since vascular occlusion is one of the proposed aetiological mechanisms of SSNHL, the hypothesis that a diet rich in saturated fat may contribute to sudden hearing loss by disrupting blood flow to the cochlear artery is plausible.^3^^,^^22^ A temporal bone histopathology study by Merchant *et al*. identified atrophy in the stria vascularis and fibrosis in cochlear arterioles in some cases of idiopathic SSNHL.^3^ These findings underscore the importance of vascular factors in the pathogenesis of SSNHL.

The finding that dietary fibre intake was markedly lower in SSNHL patients (17.37 g vs. 25.50 g; *p* < 0.001) is significant. Gut microbial fermentation of dietary fibre produces short-chain fatty acids with anti-inflammatory properties.^23^ The concept of the “gut–inner ear axis” has gained importance in recent years. A Mendelian randomisation study by Wang *et al*. published in 2024 demonstrated significant causal associations between six specific gut microbiota species and SNHL.^11^ Low fibre intake may promote dysbiosis, enhancing systemic inflammation and potentially impairing cochlear physiology.^24^ Our findings suggest that inadequate fibre intake may be a potential risk factor for SSNHL through the gut microbiota.

The lower iron and zinc intakes in the SSNHL group are also noteworthy. Zinc is an essential mineral for cochlear function and serves as a cofactor for enzymes such as carbonic anhydrase.^25^ The findings of our study suggest that inadequate zinc intake may play a role in the pathogenesis of SSNHL.

The study by Wang *et al*. also demonstrated that SNHL risk increased when DII score exceeded 5.15, although this study did not differentiate by frequency.^11^ In our study, the moderate discriminative power of DII in predicting SSNHL in ROC analysis (AUC: 0.690) indicates that it cannot serve as a standalone diagnostic tool in clinical practice but may be a useful parameter in risk assessment.

Our study has several limitations. First, due to the case–control design, causality cannot be established; only associations can be demonstrated. The possibility that patients may have altered their dietary habits after SSNHL could lead to recall bias; however, this effect was minimised by querying diet for the 12-month period prior to SSNHL. Second, the sample size is relatively small (*n* = 62); larger studies are needed to confirm the findings. Third, other potential confounding factors such as genetic factors, stress levels and physical activity were not fully controlled. Finally, the study is single-centre, and the generalisability of the results is limited.

**What is already known on the subject**

- Sudden sensorineural hearing loss (SSNHL) is an otologic emergency with a multifactorial aetiology, in which inflammatory mechanisms may play a role
- Dietary inflammatory potential, measured using the Dietary Inflammatory Index (DII), reflects the overall pro- or anti-inflammatory effect of dietary patterns

**What this paper adds to our understanding**

- In this case–control study, patients with SSNHL had significantly higher DII scores compared with healthy controls, indicating a more pro-inflammatory dietary profile
- Higher intake of saturated fat and cholesterol and lower intake of omega-3 fatty acids and dietary fibre were observed among SSNHL patients
- These findings support a potential association between pro-inflammatory dietary patterns and the risk of SSNHL
- Dietary strategies aimed at reducing systemic inflammation may represent a modifiable factor in the broader risk profile of SSNHL

## Conclusion

This study demonstrated that patients with SSNHL exhibited significantly higher DII scores than healthy controls, indicating a more pro-inflammatory dietary profile. These findings suggest a potential association between dietary inflammatory potential and the risk of SSNHL. Our results contribute to the growing body of evidence linking systemic inflammation and dietary patterns with auditory health. In this context, dietary inflammatory status may represent a modifiable factor within the broader risk profile of SSNHL. Although causality cannot be inferred due to the case–control design, these findings support the hypothesis that pro-inflammatory dietary patterns may play a role in the pathophysiological environment associated with SSNHL. From a public health perspective, dietary strategies aimed at reducing systemic inflammation may represent a potential avenue for risk reduction and future preventive approaches. Further prospective studies are required to clarify this relationship.

## Data Availability

The datasets generated during and/or analysed during the current study are available from the corresponding author on reasonable request.

## Table 1 Long description

Demographic and anthropometric characteristics are reported for a sudden hearing loss group with 30 participants and a control group with 32 participants, along with p values. Age in years is 42,60 plus or minus 12,47 in the sudden hearing loss group and 42,25 plus or minus 13,33 in the control group, with p value 0.915. Sex is listed as counts and percentages: males are 18 at 60,0 percent in the sudden hearing loss group and 17 at 53,1 percent in the control group; females are 12 at 40,0 percent and 15 at 46,9 percent, with p value 0.585. Body mass index in kilograms per meter squared is 24,31 plus or minus 4,11 in the sudden hearing loss group and 23,97 plus or minus 3,61 in the control group, with p value 0.731.

Navigate back to Table 1.

## Table 2 Long description

Mean plus or minus standard deviation values are compared between a sudden hearing loss group with 30 participants and a control group with 32 participants, with a p value for each parameter. DII score was 2.27 plus or minus 1.21 in the sudden hearing loss group and 1.46 plus or minus 1.14 in controls, p 0.01. Energy was 2251.59 plus or minus 294.28 kilocalories versus 2076.49 plus or minus 316.11, p 0.028; carbohydrate was 306.33 plus or minus 54.02 grams versus 277.34 plus or minus 43.71, p 0.023; protein was 73.08 plus or minus 12.66 grams versus 67.82 plus or minus 12.52, p 0.106. Total fat was 81.55 plus or minus 17.88 grams versus 77.32 plus or minus 16.52, p 0.31 with an asterisk; saturated fat was 28.61 plus or minus 7.83 grams versus 23.03 plus or minus 8.85, p 0.035 with an asterisk; MUFA was 33.98 plus or minus 7.32 grams versus 35.40 plus or minus 10.24, p 0.532. Cholesterol was 355.67 plus or minus 90.27 milligrams versus 257.66 plus or minus 98.91, p less than 0.001; omega 3 was 0.65 plus or minus 0.45 grams versus 1.00 plus or minus 0.63, p 0.027 with an asterisk; omega 6 was 16.87 plus or minus 5.42 grams versus 17.38 plus or minus 5.49, p 0.646 with an asterisk. Vitamin A was 948.33 plus or minus 308.55 micrograms versus 709.38 plus or minus 195.15, p 0.004 with an asterisk; vitamin C was 93.17 plus or minus 17.59 milligrams versus 80.56 plus or minus 19.47, p 0.01; vitamin E was 10.18 plus or minus 2.59 milligrams versus 7.45 plus or minus 2.66, p less than 0.001. Vitamin B6 was 1.51 plus or minus 0.27 milligrams versus 1.49 plus or minus 0.25, p 0.771; vitamin B12 was 4.81 plus or minus 0.93 micrograms versus 5.77 plus or minus 2.46, p 0.326 with an asterisk; folate was 334.33 plus or minus 104.34 micrograms versus 215.0 plus or minus 100.25, p less than 0.001. Iron was 15.47 plus or minus 4.18 milligrams versus 19.59 plus or minus 4.56, p less than 0.001 with an asterisk; magnesium was 309.17 plus or minus 47.92 milligrams versus 266.88 plus or minus 58.44, p 0.03; zinc was 12.03 plus or minus 3.15 milligrams versus 13.53 plus or minus 2.65, p 0.018 with an asterisk.

Navigate back to Table 2.
